# Impact of interprofessional collaborative practice in palliative care on outcomes for advanced cancer inpatients in a resource-limited setting

**DOI:** 10.1186/s12904-022-01121-0

**Published:** 2022-12-29

**Authors:** Pitchayapa Pornrattanakavee, Tassaya Srichan, Kasan Seetalarom, Siriwimon Saichaemchan, Nittha Oer-areemitr, Naiyarat Prasongsook

**Affiliations:** 1 Division of Medical Oncology, Department of Medicine, Phramongkutklao Hospital and College of Medicine, Bangkok, Thailand; 2 Division of Nursing, Department of Medicine, Phramongkutklao Hospital, Bangkok, Thailand; 3 Division of Pulmonary and Critical Care Medicine, Department of Medicine, Phramongkutklao Hospital and College of Medicine, Bangkok, Thailand

**Keywords:** Advanced cancer inpatients, Interprofessional collaborative practice, Team-based approach, Palliative care, Specialty palliative care, Palliative care team, Quality of life (QoL), Re-admission, Anxiety, Depression

## Abstract

**Background:**

Palliative care for patients with advanced cancer improves suffering symptoms, and quality of life (QoL). However, routine implementation of palliative care by specialty palliative care consultation is still an unmet need among in-patients with advanced cancer. Our study aim is to evaluate the effectiveness of a team-based approach on QoLs and readmission rate when compared to routine practice by among medical oncologists.

**Methods:**

This study was a prospective, Quasi-Experimental design. In-patients with advanced cancer were non-randomly assigned to receive palliative care service by team-based approach or medical oncologists only. The primary endpoint was QoL. The secondary endpoint was the readmission rate at 7 and 30 days of hospital discharge.

**Results:**

One hundred twenty-two in-patients were enrolled. In-patients who were assessed by a team-based approach had significantly improved change scores of subjective well-being (SWB) when compared to another group (∆ SWB: -1 [-19 – 11] vs 0 [-9 – 15], *p-value* = 0.043). Furthermore, patients who were assessed under a team-based approach had significantly decreased in terms of readmission rate at 7 days of hospital discharge (4.92% in the team-based approach group vs. 19.67% in the medical oncologist group, *p-value* = 0.013).

**Conclusions:**

Interdisciplinary collaboration is the key to success in establishing goals of care, which are supporting the best possible QoL and relieving suffering symptoms for those in-patients with advanced cancer. Furthermore, the readmission rate at 7 days of hospital discharge was significantly reduced by a team-based approach. Therefore, comprehensive palliative care assessment by interprofessional collaborative practice is required.

**Trial Registration:**

Thai Clinical Trials Registry (TCTR): number 20200312001. Date of first registration on 09/03/2020.

**Supplementary Information:**

The online version contains supplementary material available at 10.1186/s12904-022-01121-0.

## Background

Integration of palliative care in cancer patients improve oncology outcomes, including suffering symptoms, quality of life, and survival. More than 40 million people each year are expected to be needed for palliative care [1]. Furthermore, most of them (78%) live in low- and middle-income countries. However, only 14% of those people will be received comprehensive palliative care [1] due to limited-resources available for palliative care specialists particularly.

Systematic review demonstrated that the best outcomes with palliative care was provided by an interprofessional palliative care team and should be initiated within 8 weeks of diagnosis [2]. For example, a meta-analysis results showed a 14% increase in 1-year survival, and median overall survival benefit of 4.56 months in patients with advanced cancer who were received palliative care by outpatient specialty palliative care team [3]. Moreover, quality of life and cost-effectiveness were improved significantly. Regarding to recent data, comprehensive palliative care in patients with advanced cancer by interprofessional palliative care team can be favorably impact health systems and cost-effective [4, 5].

Multidisciplinary team including board-certified palliative care physicians, advanced practice nurses, physician assistants, nurses, dieticians, social workers, and pharmacists is required for providing direct care to patients, families, caregivers [2]. In fact, the most of inpatients with advanced cancer who are needed with palliative care approach in clinical practice are managed by medical oncologist alone, which palliative and oncologic aspects of care might have an inferiority for placing the entire burden on an interprofessional team approach. Therefore, a novel model of palliative care service delivery requires to be developed in limited-resources. In this study, we designed a novel model for the inpatient hospital setting, which was a palliative care nurse and medical oncology co-working model of care.

The objective of this study was to obtain the effect of the palliative care nurse and medical oncology co-working model on quality of life of inpatients with advanced cancer, and readmission rate at 7 days and 30 days of hospital discharge.

## Patients and methods

### Study design and patient selection

This study is a prospective, single institute, non-randomized interventional study design (Quasi-Experimental design) investigating the effect of interprofessional cooperative practice in palliative care on oncologic, and palliative outcomes in advanced cancer inpatients.

The trial was conducted according to ICH-GCP guidelines and the Declaration of Helsinki. The study protocol was approved by Institutional Review Boards, Phramongkutklao Hospital and College of Medicine, Royal Thai Army Medical Department. All patients provided written informed consent for trial participation. It is registered under Thai Clinical Trials Registry (TCTR)#20,200,312,001. Date of first registration was March 9, 2020.

All patients with advanced stage of solid cancer ≥ 18 years who were hospitalized due to symptomatic management, any ECOG performance status were eligible. In addition, patients who were not received any specific cancer treatment, and those patients who were able to understand and fill the questionnaires were included. Patients with hematologic malignancies were excluded. Admission criteria were patients with issues relating to physical symptoms (such as cancer pain, dyspnea, or multiple physical problems), psychological distress, complex psychosocial care needs that were difficult to address in the usual care setting, and complex end-of-life care. Readmission criteria within 30 days of a discharge from our study were 1) complication related to prior admitting treatment, or complication occurred after discharge but was directly related to the previous admission, 2) recurrence of disease/medical conditions, 3) additional therapy for worsening medical conditions, 4) unrelated new conditions/ diagnosis. All enrolled patients were followed until either discharge to home, discharge to nursing home, or death at hospital, depending on their medical conditions, availability of care givers, and their socioeconomic issues.

### Treatment

**Fig. 1:**
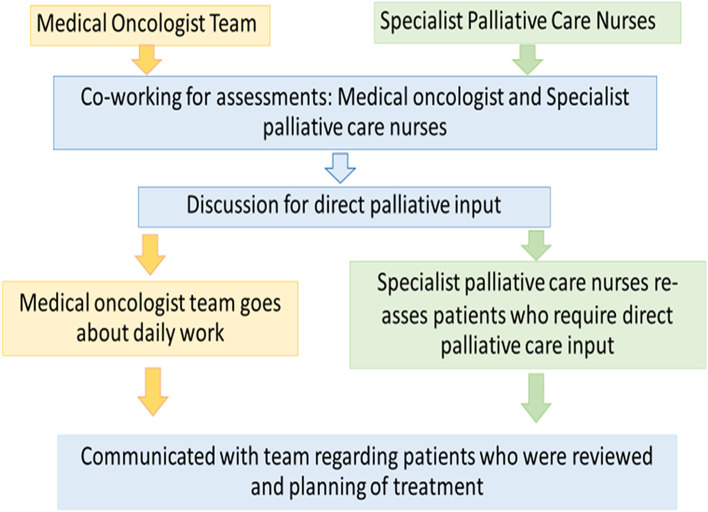
Schema of co-working for palliative care between specialist palliative care nurses and medical oncologists as interprofessional collaborative team

Eligible patients were non-randomly assigned to received palliative care by either interprofessional collaborative team, including specialist palliative care nurses and medical oncologists or medical oncologist alone, depending on the working schedules and availability of palliative care nurses. The palliative care team consists of one palliative care nurse, one nurse practitioner, and one medical oncologist. Palliative care physician by training is not available in our institute. Palliative care nurse and nurse practitioner made assessment and evaluation of physical, emotional, and social aspects of the patient’s well-being. Then, the communication with team (palliative care nurse, nurse practitioner, and medical oncologist) regarding patients who were reviewed was performed for planning of treatment. All in-patients with advanced cancer were assessed and received treatment for suffering symptoms by medical oncologists. In-patients with advanced cancer who were assigned to receive palliative care by interprofessional collaborative team were evaluated and received treatment for physical-, psychological-, and spiritual aspects by both specialist palliative care nurse and medical oncologist. Cancer pain and other symptoms of discomfort were mainly treated by medications (e.g.strong opioid for cancer pain, and dyspnea), or palliative radiation for bone pain/ obstructive pneumonitis. The palliative care visiting programs were adapted from the National Consensus Project for Quality Palliative Care [6] by using medical records and questionnaire for assessment at 1 day and 7 days after hospitalization. Both physical and psychological aspects were accessed via through medical records, and questionnaires, respectively. (Fig. 1, Supplementary Fig. 1). At discharge, specialist palliative care nurse provided health education about home care for the terminally ill to the primary caregiver. Home care follow up after discharge was not included in this study. However, home care visit was offered for patients who wish to die at home due to limited resources.

### Patient-reported measurements

For psychological aspects, we used Thai-Hospital Anxiety and Depression Scale (Thai-HADS) for assessment. The Thai-HADS is composed of 14-items, which it is divided into 2 subscales such as symptoms of anxiety scores and depression scores, each of which scores a symptom severity between a 0 and 21. For each item, a score of 0 typically indicates no distress, or no anxiety, while a higher score is indicative of higher distress/ or anxiety. If score higher than 7 indicates clinically significant of distress/ or anxiety.

Health-related quality of life was measured by using the Thai- Functional Assessment of Cancer Therapy- General (Thai-FACT-G) scale. The main purpose of Thai FACT-G is to evaluate multiple dimensions of quality of life such as physical well-being, social/family well-being, emotional well-being and functional well-being, which scores a well-being between a 0 and 108. The higher score is indicative of improvement of quality of life.

### Objectives and end points

The primary objective of the study was to compare the quality of life (QoL) between advanced cancer in-patients who were received palliative care by interprofessional collaborative team and among medical oncologists. The secondary objective was to compare the re-admission rate at 7 days and 30 days after hospital discharge between two groups.

### Sample size calculation

Regarding to the previous pilot study results [7], our hypothesis was that team-based approach was associated with better outcomes in QoL, and 10% shorter hospital length of stay, and readmission rate. Therefore, the calculated sample was 60 patients in each arm.

### Statistical analysis

Descriptive statistics were used to estimate the frequencies, means, and standard deviation of the study variables. Differences between two groups were analyzed by using Chi-square test or Fisher’s exact test. Paired T- test was used for analysis of difference in mean for Thai -FACT – General, Thai-HADS score on each group. Mann–Whitney U test was used for analysis of difference in mean for Thai-FACT-General, Thai- HADS score between two groups. For all other tests, the alpha was set to 0.05 (two-sided). All statistical analyses were performed using SPSS, software, version 22. Data cutoff date was January 14, 2021 due to reaching the planned sample size. For all outcomes, the intention-to-treat analysis was conducted.

## Results

### Patients and treatment

Between March and December 2020, we enrolled 122 patients who were diagnosed with advanced solid cancer without receiving any specific cancer treatment, and were hospitalized for symptomatic treatment in Phramongkutklao Hospital. Sixty-one patients were received palliative care treatment by specialist palliative care nurse and medical oncologist. Another 61 patients were evaluated and treated by medical oncologists. The baseline characteristics were similar between two groups, except metastatic site at liver and cancer pain aspect (Table 1). The majority were enrolled with male (68%), primary lung cancer (28.7%), and cancer pain (58.1%). The median age of patients who were received palliative care treatment by interprofessional collaborative team, and medical oncologist alone were 63, and 59 years-old, respectively. (Table 1).

**Table 1:** Baseline characteristics of the study population

	Professional Collaborative Team (*n* = 61)	Medical Oncologists (*n* = 61)	Total	*p-value*
	n (%)	n (%)	n(%)	
**Gender**				0.846
Male	42 (68.85)	41 (67.21)	83 (68.03)	
Female	19 (31.15)	20 (32.79)	39 (31.97)	
**Age**				
Median (Min—Max)	63 (20—88)	59 (22—81)		0.862‡
**date admit**				
Median (Min—Max)	14 (7—127)	11 (7—45)		0.012‡
**Type of Cancer**				
Primary Lung Cancer	14 (22.95)	21 (34.43)	35 (28.68)	0.161
Hepatocellular Carcinoma	11 (18.03)	3 (4.92)	14 (11.47)	0.023
Esophagus/Gastric/billiary tract cancer	11 (18.03)	12 (19.67)	23 (18.85)	0.817
Head and Neck Cancer	4 (6.56)	9 (14.75)	13 (10.65)	0.142
Colon Cancer	4 (6.56)	6 (9.84)	10 (8.19)	0.509
Genitourinary Cancer	6 (9.84)	2 (3.28)	8 (4.91)	0.272†
Breast Cancer	4 (6.56)	4 (6.56)	8 (6.55)	1.000
Musculoskeletal Cancer	4 (6.56)	1 (1.64)	5 (4.09)	0.365†
Two primary cancer	2 (3.28)	2 (3.28)	4 (3.27)	1.000
Gynecologic Cancer	1 (1.64)	1 (1.64)	2 (1.63)	1.000†
**Brain Metastasis at Admission**	6 (9.84)	9 (14.75)	15 (12.29)	0.408
**Liver Metastasis at Admission**	27 (44.26)	13 (21.31)	40 (32.78)	0.007
**Type of Treatment**				
Chemotherapy	38 (62.30)	38 (62.30)	76 (62.3)	1.000
Surgery	19 (31.15)	18 (29.51)	37 (30.3)	0.844
Immunotherapy/Targeted	7 (11.48)	10 (16.39)	17 (13.4)	0.433
Hormonal therapy	5 (8.20)	1 (1.64)	6 (4.9)	0.207†
**Type of Problems**				
Pain	45 (73.77)	26 (43.33)	71 (58.19)	0.001
Dyspnea	21 (34.43)	29 (47.54)	50 (40.98)	0.141
Fatigue	23 (37.70)	16 (26.23)	39 (31.96)	0.174
Abdominal Pain	25 (40.98)	17 (27.87)	42 (34.42)	0.127
Nausea	7 (11.48)	12 (19.67)	19 (15.57)	0.212
Bedsore	1 (1.64)	-	1 (0.8)	1.000†

Chi-Square test

^†^Fisher's exact test

^‡^Mann–Whitney U test

### Psychological outcomes

For Thai-HAD scores at baseline (day 1 of hospitalization), the majority of all enrolled patients had no anxiety, and depression, which the percentage were 59%, and 53%, respectively. Almost twenty percent of all enrolled patients had significant anxiety. Approximately one-fourth of all enrolled patients were facing with depression at day 1 of hospitalization. The median of anxiety-, and depression subscale at baseline were not different between two groups (Table 2). The median anxiety score for professional collaborative team group and medical oncologist group were similar, which score was 6. Moreover, the median depressive score for professional collaborative team group and medical oncologist group at baseline were not statistically significant different, which depressive scores were 6, and 9, respectively (*p-value* = 0.13) (Table 2).

**Table 2:** Thai-Hospital Anxiety and Depression Scale (Thai-HADS) at baseline (day 1 of hospitalization) between the two groups

	Professional Collaborative Team (*n* = 61)	Medical Oncologists (*n* = 61)	Total	*p-value*
	n (%)	n (%)	n (%)	
**Anxiety**				0.961
Absent	36 (59.02)	36 (59.02)	72 (59)	
Yes, but normal	13 (21.31)	14 (22.95)	27 (22.1)	
Yes, abnormal	12 (19.67)	11 (18.03)	23 (18.8)	
Median (Min—Max)	6 (0—16)	6 (0—19)		0.734‡
**Depression**				0.062
Absent	39 (63.93)	26 (42.62)	65 (53.2)	
Yes, but normal	8 (13.11)	13 (21.31)	21 (17.2)	
Yes, abnormal	14 (22.95)	22 (36.07)	36 (29.5)	
Median (Min—Max)	6 (0—18)	9 (0—21)		0.137‡

Chi-Square test

Significant if *p* < 0.05

^‡^Mann–Whitney U test

**Fig. 2:**
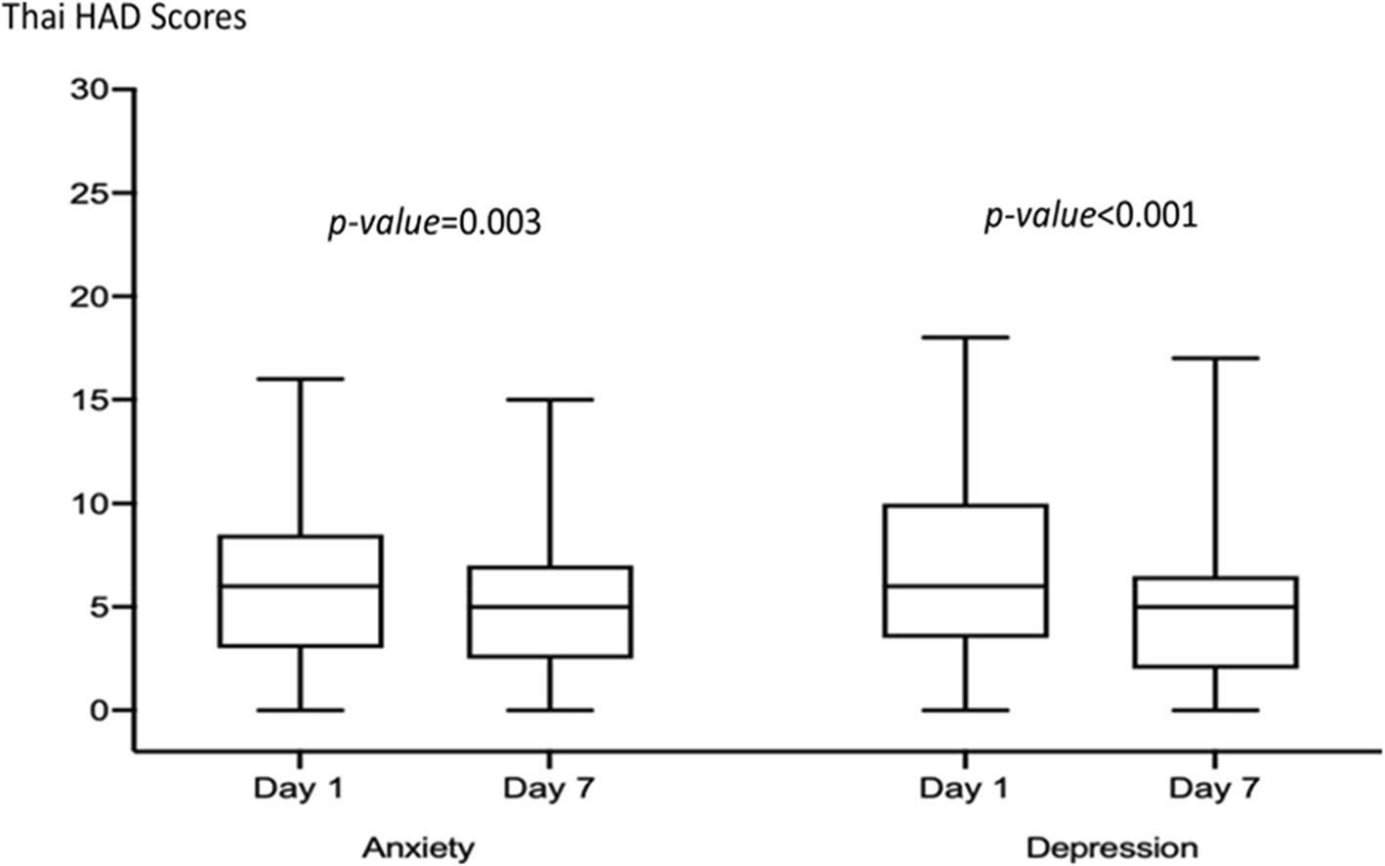
Thai-Hospital Anxiety and Depression Scale (Thai-HADS) at days 1 and 7 of hospitalization in a professional collaborative team

For Thai-HAD scores at 7 days of hospitalization, the median anxiety score for patients with professional collaborative team group was 5, which score was lower than median anxiety score at day 1 of hospitalization significantly (*p-value* = 0.003). Additionally, the median depressive score for patients with professional collaborative team group was 5, which score was lower than median depressive score at day 1 of hospitalization significantly (*p-value* < 0.001) (Fig. 2). The median anxiety score for patients with medical oncologist group at day 7 of hospitalization was 6, which median score was not statistically significant different from day 1 (*p-value* = 0.06). Meanwhile, the median depressive score for patient with medical oncologist group at day 7 was lower than score at day 1 of hospitalization significantly, which median score was 6 at day 7 when compared to score of 9 at day 1 of hospitalization (*p-value* = 0.006) (Table 3). However, the difference in the median of anxiety and depressive scores from day 1 to day 7 was not statistically significant different between two groups (Supplementary Table 1).

**Table 3:** Thai Hospital Anxiety and Depression Scale (Thai-HADS) at days 1 and 7 of hospitalization on each group

	Day 1	Day 7	*p-value*
**Professional Collaborative Team**			
Anxiety			
median (min—max)	6 (0—16)	5 (0—15)	0.003
Depression			
median (min—max)	6 (0—18)	5 (0—17)	< 0.001
**Medical Oncologists**			
Anxiety			
median (min—max)	6 (0—19)	6 (0—19)	0.068
Depression			
median (min—max)	9 (0—21)	6 (0—19)	0.006

Wilcoxon Signed Ranks test

Significant if *p* < 0.05

### Quality of life outcomes

There was similar in Thai FACT-G score including physical-, social-, emotional-, and functional well-being scores at baseline (day 1 of hospitalization) between two groups (*p-value* = 0.55) (Supplementary Table 2).

**Fig. 3:**
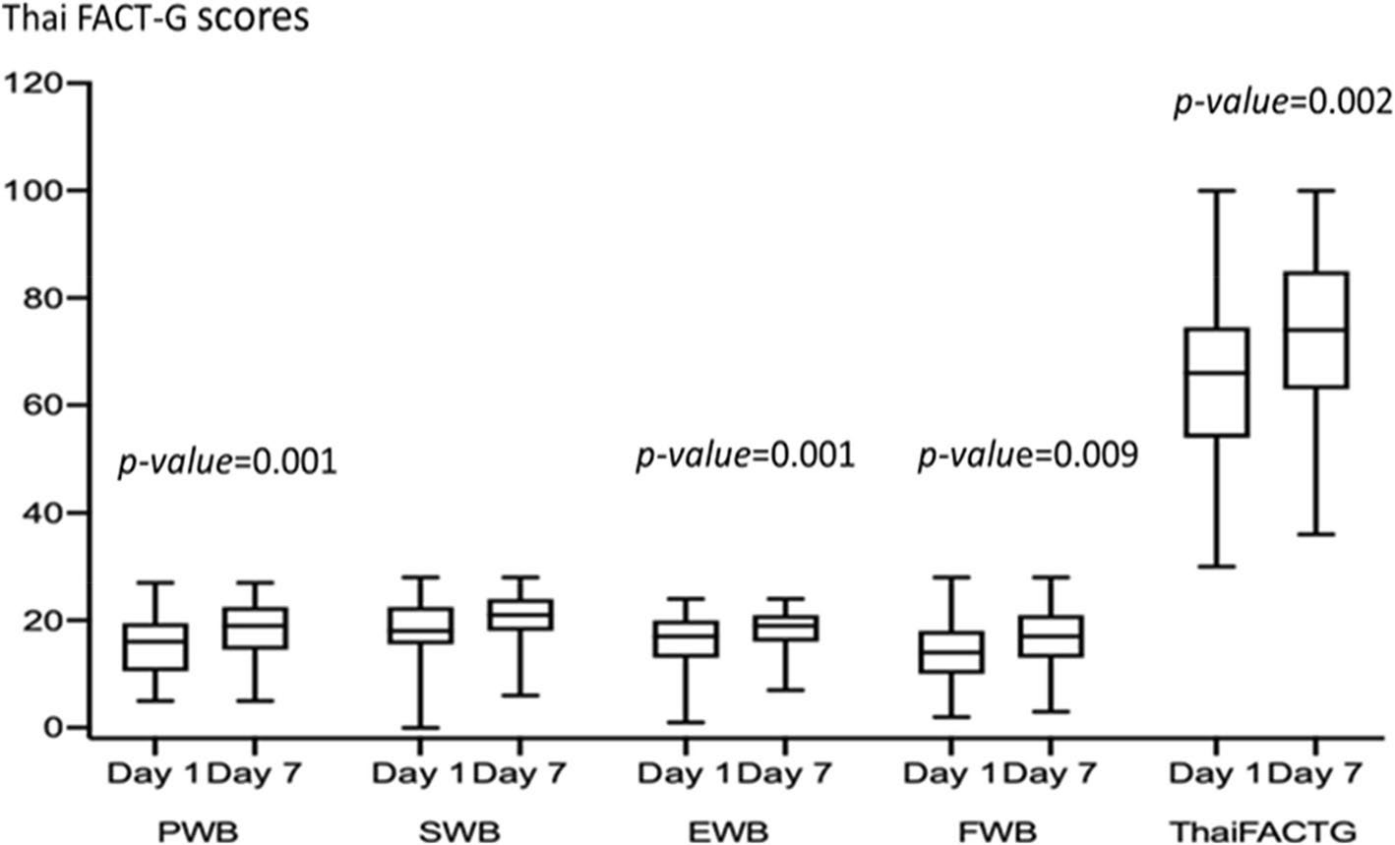
Thai-Functional Assessment of Cancer Therapy-General (Thai FACT-G) scale at days 1 and 7 of hospitalization in the interprofessional collaborative team

For Thai FACT-G scores at 7 days of hospitalization, the median Thai FACT-G score for patients with professional collaborative team group was higher than scores at day 1 of hospitalization significantly including physical-, emotional-, functional well-being scores (Fig. 3). The median Thai FACT-G scores for patients with medical oncologist group at day 7 of hospitalization was not statistically significant different from day 1 (*p-value* = 0.16), except physical-, and functional well-being subscale at day 7 were higher than day 1 of hospitalization significantly (Supplementary Table 3). The difference in the median of Thai FACT-G scores from day 1 to day 7 was not statistically significant different between two groups, except the difference in the median of social well-being subscale from day 1 to day 7 was significantly improved for patients with professional collaborative team group, when compared to patients with medical oncologist group (*p-vale* = 0.043) (Supplementary Table 4, Supplementary Fig. 2).

### Quality of care outcomes

Re-admission rate at 7 days after hospital discharge was statistically significant reduced in patients with interprofessional collaborative team group when compared to patients with medical oncologist team group, which re-admission rate were 4.9%, and 19.7% (*p-value* = 0.013), respectively. Twelve of 61 patients with medical oncologist team were re-hospitalized at 7 days after hospital discharge, and only 3 of 61 patients with interprofessional collaborative team group. However, there was not statistically significant different in re-admission rate at 30 days after hospital discharge between two group, which re-admission rate were 32.8% in patients with interprofessional collaborative team group, and 40.9% in patients with medical oncologist team (*p-value* = 0.34). Both groups were still alive at 30 days after hospital discharge, which the percentage was approximately 80%. The median duration of hospitalization was longer in patients with interprofessional collaborative team group than patients with medical oncologist team significantly, which was 14 days (7–127 days), and 11 days (7–45 days) (*p-value* = 0.01) (Supplementary Table 5).

## Discussion

The aim of this study is to endorse that advanced solid cancer in-patients experience the best possible quality of life from palliative care in resource limited health care environment. Regarding to complex mechanisms and clinical presentations of suffering symptoms, including its physical, psychological, and spiritual aspects, multidisciplinary team approach is required for providing the effective palliative care. Moreover, the discordance between patients-reported and physician-documented symptoms was reported [8]. One study shown that compared 58 questionaries completed by advanced cancer patients and their paired physician completed in medical records. The results showed that pain assessment was high concordance (96%), but psychological and other aspects were discordance, which is associated with poor QoL, and distress [6, 8].

In our limited resource condition, interprofessional team approach for this study was co-working in palliative care between specialist palliative care nurses, practitioner nurse, and medical oncologists. In fact, mood disorders are major contributors to morbidity in patients with advanced cancer [9]. We considered using Thai-Hospital Anxiety and Depression Scale (Thai-HADS), and Thai-Functional Assessment of Cancer Therapy-General (Thai FACT-G) scale as main measurement. The Thai-Hospital Anxiety and Depression Scale (Thai-HADS) is the most reliability and validity for mood assessment in palliative care setting. Moreover, the Thai-Functional Assessment of Cancer Therapy-General (Thai FACT-G) is the best tool for comprehensive quality of life assessments, including its physical, social, emotional, and functional well-beings [2]. Furthermore, palliative care assessment for hospitalized advanced cancer patients in the Netherland evaluated the quality of life by using EQ5D scores, which the questionnaires provide a simple, generic questionnaire for use in clinical and economic appraisal and population health surveys [10].

The team approach from our study explored the effect of having specialist palliative care nurses, practitioner nurse, and medical oncologists in the palliative care team, which team-based approach significantly improved anxiety-, and depressive subscale at day 7 of hospitalization, and clinical meaningfully improved quality of life in social well-being subscale. Our study results were similar to previous studies. For example, one randomized controlled trial showed improvement of depressive subscale significantly in advanced non-small cell lung cancer patients who were assessed palliative care by multidisciplinary team approach (*p-value* = 0.01) [2]. In addition, the ENABLE trial shown that cancer patients who were received palliative care by specialist palliative care team had lower incidence of depression than patients who were received assessment by physicians (*p-value* = 0.02) regarding to focusing on coping skills and psychosocial concerns from specialist palliative care professionals [10]. Another randomized controlled trial demonstrated that early palliative care by specialist palliative care team significantly improved depressive symptoms in advanced stage cancer patients (*p-value* = 0.01), and depressive symptom was associated with mortality [11].

The rationale of our co-working model of care is primarily care for cancer patients and their family. Therefore, our interprofessional collaborative team needs to recognize the relationship between cancer patients, their family, and their primary physician. Medical oncologist was required to provide continuum of care for cancer patients to relief from physical, emotional sufferings. Palliative care nurse actively participated in the process of conducting a comprehensive assessment of cancer patients and their family. Importantly, our interprofessional collaborative team met regularly to collaboratively review and plan of management for cancer patients and their family. Therefore, the results from this study confirmed that the need for more comprehensive assessment of patient’s quality of life from team-based approach provided better in palliative care outcomes in patients with advanced stage of cancer. Furthermore, this co-working model of palliative care for patients with advanced cancer leads us to develop an interprofessional collaborative practice competencies, which were collaboration, communication, responsibilities and roles of team member, and ethics. Additionally, one study demonstrated a positive correlation between quality of life of advanced cancer patients and their survival [7, 12–14]. Therefore, we hypothesize that improvement of mood and quality of life from palliative care in advanced cancer patients may prolong their survival. Further study should be explored.

There are a few studies focusing on readmissions for cancer patients. However, the re-admission rate is a crucial balancing measure to indicate a quality of palliative care and continuity of care, and healthcare resource allocation [15]. Our study found that readmission rate was significantly reduced in patients who were received palliative care by team-based approach significantly. However, the median length of stay for patients who were assessed by our team-based approach were longer than patients who were assessed by medical oncologist team, which the median duration of hospital stay were 14 days (7–127 days), and 11 days (7-45 days), respectively. In fact, discharge decision in our institute was primarily made by primary physicians and based on medical conditions, preparedness for caregiving in palliative care at home, and preferences of patients and their relatives. Therefore, patients who were assessed by our team-based approach with longer in length of hospital stay might indicate a relatively unstable conditions, unpreparedness of caregiving at home, or unwillingness for hospital discharge. Regarding to non-randomized study design, our provocative results would seem to conflict with existing theories that readmission rate is indication of quality issue related to shortened length of stay. In addition, patients who were assessed by our team-based approach with longer in length of hospital stay might indicate a relatively unstable condition.

Only approximately 10% on each group died during their admission for receiving palliative care, while the population-based cohort study from Taiwan shown that the majority of hospitalized cancer patients for palliative care (59%) died during their first admission [16]. This result may indicate that an early palliative care is associated with reduction of mortality rate during admission, which early palliative care in our study was defined regarding the inclusion criteria that an integration of palliative care should be provided in the earlier part of initial clinical symptoms for those advanced cancer patients who were not received any specific treatment. Moreover, this study demonstrated that poor communication with patients and their family, lack of home care follow-up, inadequate outpatient follow-up and care coordination may attribute to increase readmission rate and mortality [16].

This study had several limitations. First, the study design was non-randomization, therefore selection bias could be occurred due to an error in the procedure used to select target populations. Moreover, it could potentially occur self-selection bias because it is likely that their motivation for participation into interprofessional collaborative team group. Second, generalizability as this study was conducted in an institution. Third, there was short-term follow up of this study. Fourth, there was not ideally multidisciplinary team-based approach regarding limited resource in human reason. Fifth, we did not distinguish subclassification of advanced stage to terminal stage of disease. To address the limitations, the randomization method eliminates the selection bias, and balances the groups with respect to many confounding variables. Therefore, further randomized study is planned. Furthermore, multicenter study and longer follow up time are required.

## Conclusion

Our study concluded that co-working and the communication between specialist palliative care nurses and medical oncologists as team-based approach is considered to be a key factor for effective interprofessional collaboration in a resource limited setting. Advanced cancer patients who were assessed palliative care with team-based approach improved their either quality of life or psychological aspects, and significantly reduce readmission rate at 7 days after hospital discharge. The results may help inform policy discussions to improve the quality of palliative care and to reduce the readmission rate among advanced cancer inpatients.

## Supplementary Information

**Additional file 1:** **Supplementary Figure 1.** Schema for palliative care in clinical practice by medical oncologists. **Supplementary Figure 2.** Comparison of the difference in median of Thai-Functional Assessment of Cancer Therapy-General (Thai FACT-G) scale from day 1 to day 7 of hospitalization between Professional Collaborative Team group and Medical Oncologist group. **Supplementary Table 1.** Comparison of the difference in median of Thai-Hospital Anxiety and Depression Scale (Thai-HADS) from day 1 to day 7 of hospitalization between Professional Collaborative Team group and Medical Oncologist group. **Supplementary Table 2.** Thai-Functional Assessment of Cancer Therapy- General (Thai FACT-G) scale at baseline. **Supplementary Table 3.** Thai-Functional Assessment of Cancer Therapy-General (Thai FACT-G) scale at day 1 and day 7 of hospitalization on each group. **Supplementary Table 4.** Comparison of the difference in median of scale from day 1 to day 7 of hospitalization between Professional Collaborative Team group and Medical Oncologist group. **Supplementary Table 5.** Re-admission events (rate) at 7 days, and 30 days after hospital discharge, duration of hospitalization, and discharge status between two groups.

## Data Availability

The authors confirm that the data supporting the findings of this study are available within the article and its supplementary materials.
