# Supplementary material for: Impact of interprofessional collaborative practice in palliative care on outcomes for advanced cancer inpatients in a resource-limited setting
Source: BMC Palliat Care. 2022 Dec 29;21:229. doi: 10.1186/s12904-022-01121-0 (PMC9798714; doi:10.1186/s12904-022-01121-0)
Supplement: Supplementary file 1 — Additional file 1: Supplementary Figure 1. Schema for palliative care in clinical practice by medical oncologists. Supplementary Figure 2. Comparison of the difference in median of Thai-Functional Assessment of Cancer Therapy-General (Thai FACT-G) scale from day 1 to day 7 of hospitalization between Professional Collaborative Team group and Medical Oncologist group. Supplementary Table 1. Comparison of the difference in median of Thai-Hospital Anxiety and Depression Scale (Thai-HADS) from day 1 to day 7 of hospitalization between Professional Collaborative Team group and Medical Oncologist group. Supplementary Table 2. Thai-Functional Assessment of Cancer Therapy- General (Thai FACT-G) scale at baseline. Supplementary Table 3. Thai-Functional Assessment of Cancer Therapy-General (Thai FACT-G) scale at day 1 and day 7 of hospitalization on each group. Supplementary Table 4. Comparison of the difference in median of scale from day 1 to day 7 of hospitalization between Professional Collaborative Team group and Medical Oncologist group. Supplementary Table 5. Re-admission events (rate) at 7 days, and 30 days after hospital discharge, duration of hospitalization, and discharge status between two groups. [file 12904_2022_1121_MOESM1_ESM.docx]

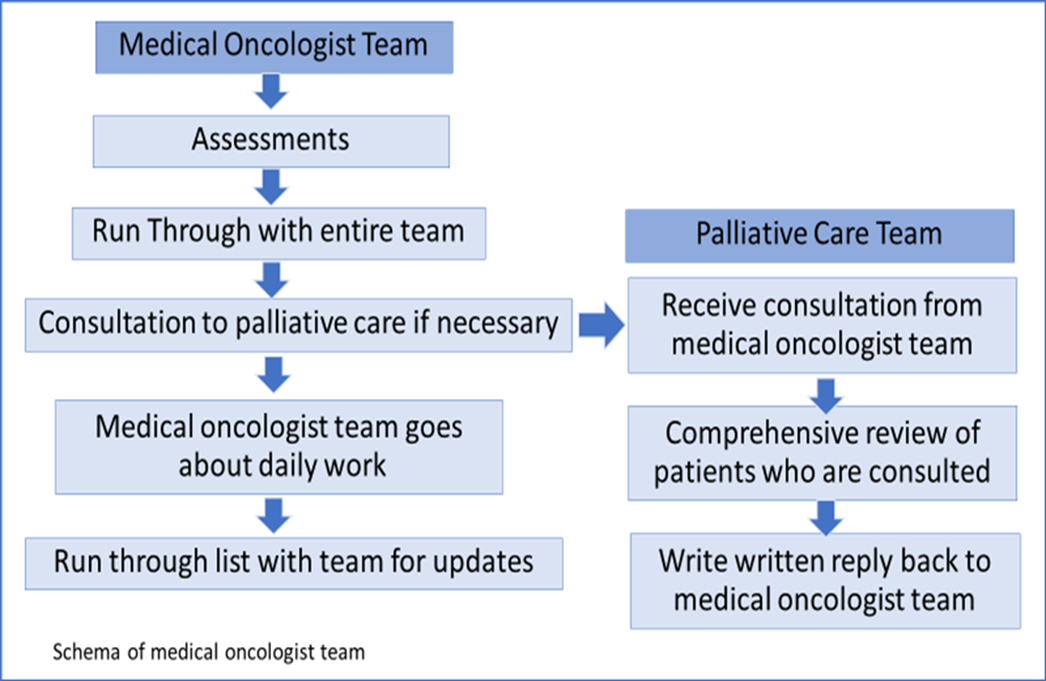
**Supplementary Figure 1.** Schema for palliative care in clinical practice by medical oncologists.

**Supplementary Figure 2.** Comparison of the difference in median of Thai-Functional Assessment of Cancer Therapy-General (Thai FACT-G) scale from day 1 to day 7 of hospitalization between Professional Collaborative Team group and Medical Oncologist group.


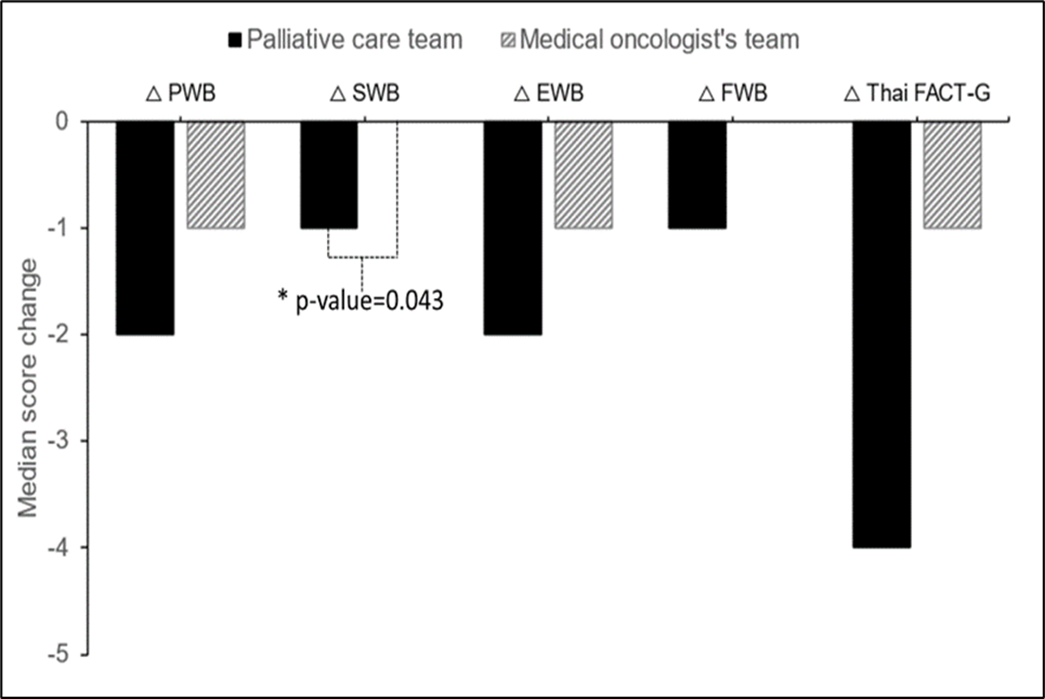


| **Supplementary Table 1.** Comparison of the difference in median of Thai-Hospital Anxiety and Depression Scale (Thai-HADS) from day 1 to day 7 of hospitalization between Professional Collaborative Team group and Medical Oncologist group | | | |
| --- | --- | --- | --- |
|  | Professional Collaborative Team (n=61) | Medical Oncologists Team(n=61) | *p-value* |
|  | n (%) | n (%) |  |
| **△ Anxiety** |  |  |  |
| median (min - Max) | 0 (-7 - 13) | 0 (-8 - 9) | 0.393‡ |
| **△ Depression** |  |  |  |
| median (min - max) | 1 (-5 - 14) | 1 (-7 - 10) | 0.622‡ |
| ‡Mann-Whitney U test |  |  |  |
| Significant if p<0.05 |  |  |  |

**Supplementary Table 2.** Thai-Functional Assessment of Cancer Therapy- General (Thai FACT-G) scale at baseline

|  | Professional Collaborative Team (n=61) | Medical Oncologists (n=61) | *p-value* |  |
| --- | --- | --- | --- | --- |
|  | median (min - max) | median (min - max) |  |  |
| Physical Well-Being | 16 (5 - 27) | 16 (7 - 27) | 0.967 |  |
| Social Well-being | 18 (0 - 28) | 18 (7 - 28) | 0.681 |  |
| Emotional Well -being | 17 (1 - 24) | 16 (3 - 24) | 0.665 |  |
| Functional Well-being | 14 (2 - 28) | 13 (4 - 23) | 0.451 |  |
| Thai FACT-G | 66 (30 - 100) | 60 (31 - 95) | 0.552 |  |
| Mann-Whitney U test |  |  |  |  |
| Significant if p<0.05 |  |  |  |  |

| **Supplementary Table 3.** Thai-Functional Assessment of Cancer Therapy-General (Thai FACT-G) scale at day 1 and day 7 of hospitalization on each group | | |  |
| --- | --- | --- | --- |
|  | Day 1 | Day 7 | *p-value* |
|  | median (min - max) | median (min - max) |  |
| **Interprofessional collaborative Team** |  |  |  |
| Physical Well-being | 16 (5 - 27) | 19 (5 - 27) | 0.001 |
| Social Well-being | 18 (0 - 28) | 21 (6 - 28) | 0.052 |
| Emotional Well -being | 17 (1 - 24) | 19 (7 - 24) | 0.001 |
| Functional Well-being | 14 (2 - 28) | 17 (3 - 28) | 0.009 |
| Thai FACT-G | 66 (30 - 100) | 74 (36 - 100) | 0.002 |
| **Medical Oncologists** | |  |  |
| Physical Well-being | 16 (7 - 27) | 16 (7 - 27) | 0.020 |
| Social Well-being | 18 (7 - 28) | 19 (5 - 28) | 0.320 |
| Emotional Well -being | 16 (3 - 24) | 17 (3 - 24) | 0.045 |
| Functional Well-being | 13 (4 - 23) | 13 (3 - 27) | 0.393 |
| Thai FACT-G | 60 (31 - 95) | 66 (41 - 99) | 0.106 |
| Wilcoxon Signed Ranks test | |  |  |
| Significant if p<0.05 | |  |  |

| **Supplementary Table 4.** Comparison of the difference in median of  scale from day 1 to day 7 of hospitalization between Professional Collaborative Team group and Medical Oncologist group | | | | |
| --- | --- | --- | --- | --- |
|  | Professional Collaborative Team, | Medical Oncologists, | *p-value* |  |
|  | median (min - max) | median (min - max) |  |  |
| △ Physical Well-being | -2 (-17 - 10) | -1 (-17 - 8) | 0.283 |  |
| △ Social Well-being | -1 (-19 - 11) | 0 (-9 - 15) | 0.043 |  |
| △ Emotional Well-being | -2 (-14 - 8) | -1 (-11 - 9) | 0.181 |  |
| △ Functional Well-being | -1 (-17 - 11) | 0 (-22 - 10) | 0.120 |  |
| △ Thai FACT-G | -4 (-50 - 23) | -1 (-44 - 26) | 0.134 |  |
| Mann-Whitney U test | |  |  |  |
| Significant if p<0.05 | |  |  |  |

**Supplementary Table 5.** Re-admission events (rate) at 7 days, and 30 days after hospital discharge, duration of hospitalization, and discharge status between two groups.

|  | Professional Collaborative Team (n=61) | Medical Oncologists (n=61) | Total | p-value |
| --- | --- | --- | --- | --- |
|  | n (%) | n (%) | n(%) |  |
| **Re-Admission 7 days After Discharge** | |  |  | 0.013 |
| yes | 3 (4.92) | 12 (19.67) | 15 (12.2) |  |
| no | 58 (95.08) | 49 (80.33) | 107 (87.8) |  |
| **Re-admission 30 day After Discharge** | |  |  | 0.348 |
| yes | 20 (32.79) | 25 (40.98) | 45(36.8) |  |
| no | 41 (67.21) | 36 (59.02) | 77(63.2) |  |
| **Status at 30 days** |  |  |  | 0.142 |
| alive | 48 (78.69) | 54 (88.52) | 102(83.6) |  |
| death | 13 (21.31) | 7 (11.48) | 20(16.4) |  |
| **Duration of Admission** |  |  |  |  |
| Median (Min - Max) | 14 (7 - 127) | 11 (7 - 45) |  | 0.012‡ |
| **Type of Discharge** |  |  |  | 1.000† |
| Death During Admission | 8 (13.11) | 7 (11.48) | 15(12.3) |  |
| Stay at Home | 53 (86.89) | 53 (86.89) | 106(86.8) |  |
| Admission other Hospital | - | 1 (1.64) | 1(0.9) |  |
| Chi-Square test |  |  |  |  |
| Significant if p<0.05 |  |  |  |  |
